# The return-to-work process of individuals sick-listed because of whiplash-associated disorder: a three-year follow-up study in a Danish cohort of long-term sickness absentees

**DOI:** 10.1186/1471-2458-14-113

**Published:** 2014-02-04

**Authors:** Sarah Biering-Sørensen, Anne Møller, Christian DG Stoltenberg, Jonas W Holm, Peder G Skov

**Affiliations:** 1 Department of Occupational Medicine, Køge Sygehus, Lykkebækvej 1, DK-4600 Køge, Denmark; 2 The Research Unit for General Practice, Department of Public Health, University of Copenhagen, Øster Farimagsgade 5, DK-1014 Copenhagen K, Denmark

**Keywords:** Return to work, Whiplash, Whiplash-associated disorder, Musculoskeletal disorders, Long-term sickness absence, Prognosis, Public transfer income

## Abstract

**Background:**

The chronic course of whiplash-associated disorder (WAD) has implications for both the individual and society. It has been shown that up to 50% of patients have not yet returned to work six months after a whiplash injury. We wanted to study the return-to-work (RTW) process in individuals sick-listed for more than eight weeks in six Danish municipalities. RTW in individuals sick-listed due to WAD was compared to that in those sick-listed for other musculoskeletal disorders (MSD).

**Methods:**

Information about long-term sick-listed individuals in six Danish municipalities was retrieved from an existing database. Data on public transfer income were collected and the RTW process was followed on a weekly basis. Multivariate logistic regression analysis of RTW was done four times during the first three years after the start of sick-listing.

**Results:**

One hundred and four individuals were sick-listed due to WAD and 3,204 individuals were sick-listed due to other MSDs. After 6 months, the RTW was significantly lower in the WAD group. OR for RTW in the WAD group was 0.29 (0.18–0.49) compared to the MSD group. The RTW process for both groups stabilised after two years of follow-up; 44% returned to work in the WAD group as compared to 58% in the MSD group.

**Conclusion:**

Sick-listed individuals with whiplash-associated disorder are less likely to return to work than individuals who are sick-listed because of other musculoskeletal disorders. In both groups, RTW stabilised after two years of follow-up.

## Background

Whiplash-associated disorder (WAD) is a significant health problem and it results in substantial socioeconomic costs throughout the industrialised world [1]. The term whiplash is used to describe both the acceleration-deceleration mechanism of energy transfer to the neck that results from rear-end and side-impact motor vehicle collisions *and* the variety of clinical manifestations it can cause [2,3]. Chronic symptoms after whiplash trauma such as neck pain, headache, and/or cognitive and emotional problems are well-known [4].

The chronic course of WAD has implications for both the individual and society. Previous studies have shown that between 19% and 60% of patients still have complaints six months after a whiplash injury, and 13–50% of patients are still absent from work or are unable to perform their usual activities at this time [5]. It is difficult to predict which patients will develop a chronic course, but several prognostic factors have been identified, such as high pain intensity initially [5], passive style of coping, depressed mood, fear of neck movement [6], and pain catastrophizing [7]. Implications of chronicity include significant socioeconomic costs for society such as long-term sick leave, disability pensions, and claims for compensation [8,9]. The long-term consequences of WAD have only been studied to a minor degree in Denmark, but in a study from Aarhus 12% had not returned to normal daily activity or had only returned to modified job functions one year after a whiplash injury [10]. The results of a Dutch study agreed with this finding, as 12.6% of participants had persistent work disability after one year [11]. From experiences in our daily clinical work we hypothesised that the disability among sick-listed due to WAD was higher compared to sick-listed due to other kinds of musculoskeletal diseases. Therefore the aim of the current study was to characterise the return-to-work (RTW) process after a period of long-term sick leave. We compared the process of RTW in people who were sick-listed due to WAD to the process of RTW in those who were sick-listed because of other musculoskeletal disorders (MSDs). Since the termination of a sick leave period does not always denote the recovery of the person, we preferred to use RTW as outcome measure. To our knowledge, this is the first Danish study with more than one year of follow-up regarding the occupational status of individuals who have been sick-listed because of WAD.

## Methods

### Study population

A cohort of long-term sick-listed individuals was established in six municipalities in eastern Denmark as described in detail by Stoltenberg and Skov [12]. Social workers from six municipalities registered all the individuals who had been sick-listed for at least eight weeks from 1 October 2002 to 31 December 2005. These people were defined as long-term sickness absentees [13]. The cohort consisted of 7,780 long-term sickness absentees aged 18–58 who had not received any public transfer income in the week previous to sick listing, which means that they were most likely working before inclusion. Data on public transfer income were collected from the Danish National Register of Public Transfer Payments (the DREAM database). This database registers recipients of all types of public transfer income in Denmark [14] and therefore also receipt of sickness benefits. In the Danish social security system people not at work would most likely receive some kind of public transfer income registered in the DREAM database. The Danish social security system is complex and includes dozens of different types of transfer income. The type and size of transfer income that citizens receive depend, among other things, on their status on the labour market, their membership of a union fund, as well as their reason for not being at work, such as sickness, educational activities, maternal leave, early retirement, and participating in job-qualifying activities. Data on education were collected from Statistics Denmark.

### Diagnoses

The social workers in the municipalities registered the diagnoses stated as being the cause of the sick leave, either from forms submitted by the general practitioner or from forms submitted by the sickness absentee. There were 24 different groups of diagnoses in the database, and the diagnoses were categorized in five main groups: 1) musculoskeletal disorders (back problems, muscle pain, fractures, WAD and MSD’s in general), 2) psychological disorders (depression, stress, anxiety etc.), 3) disorders related to internal medicine (cardiovascular disorders, lung disorders and diabetes), 4) cancer, and 5) others (allergy, eye disorders etc.). In this study only participants in the MSD group were included and the sub-group of MSDs sick-listed due to WAD was compared to the rest of the participants in the MSD group.

### Follow-up

Data on public transfer income were collected weekly from the DREAM database. No receipt of any public transfer income for one week was defined as RTW. Follow-up data were complete for the entire population in the first two years after onset of sick leave, but approximately a quarter of the population entered the study too late to be followed up for the full third year [12].

### Analysis

In the Danish welfare system, public benefits are highly integrated in the labour market, and as expected we found that the study population exhibited very frequent changes between ‘no public transfer income’ (RTW) and ‘public transfer income’ (not at work but sick-listed, part-time sick-listed or receiving other types of transfer income) [12]. One week of no transfer income is therefore seen as RTW, but receipt of public transfer income in the following week indicates an un-sustained RTW. Thus, the RTW rate (percentage of people back at work) was calculated continuously for every week of follow-up and presented in a simple graphic presentation, and, afterwards, the RTW-process was analysed in multivariate logistic regression models. This presentation of the RTW process has been used in intervention studies in this field of research [15,16].

The RTW process for individuals who were sick-listed because of WAD was compared with that for individuals who were sick-listed because of MSDs. Multivariate logistic regression analyses of RTW were conducted for the two groups at six months and at one, two, and three years after the start of sick leave. The MSD group served as a reference group in the analysis, which was carried out in three steps. The first step consisted of an unadjusted analysis comparing WAD versus MSD. In the second step adjustment for sex and age (continuous) was performed, and, in the third step, educational level was included (six categories based on the inherent categorisation of data from Statistics Denmark [12]).

The study was reported to The Danish Data Protection Agency. According to Danish law, research projects based on questionnaires and registers need no ethical approval.

## Results

In the cohort, 104 people were sick-listed due to WAD and 3,204 people were sick-listed due to other MSDs (26% back problems, 13% fractures and 61% unspecified MSD). Table 1 lists the main characteristics of the study population. In the group of sick-listed individuals with MSDs, 52% were women, whereas 75% were women in the WAD group. In general, people with WAD were younger than those with MSDs. Mean age for people with MSDs was 42 years, while it was 36 years in the WAD group. Individuals with WAD had a higher educational level than those with MSDs.

**Table 1:** Characteristics of the study population

**Variable**	**Category**	**WADª**	**MSD**^**b**^	**p-value (Chi-square test)**
		**N (%)**	**N (%)**	
Sex	Male	26 (25)	1526 (48)	< 0.001
	Female	78 (75)	1678 (52)	
Age	18–30 years	20 (19)	472 (15)	< 0.001
	30–39	55 (53)	873 (27)	
	40–49	18 (17)	894 (28)	
	50–58	11 (11)	965 (30)	
Education	Basic school 8–10th grade	20 (19)	1029 (33)	0.008
	General and vocational upper secondary school	6 (6)	129 (4)	
	Vocational education	56 (54)	1527 (49)	
	Short higher education	3 (3)	142 (5)	
	Medium higher education	16 (16)	238 (8)	
	Bachelor and longer higher education	2 (2)	68 (2)	

ªSick-listed due to whiplash-associated disorder.

^b^Sick-listed due to other musculoskeletal disorders.

### The RTW process

**Figure 1:**
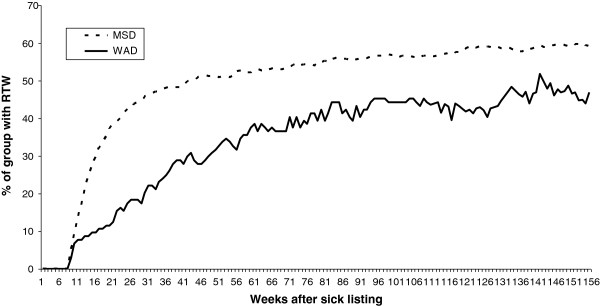
The return-to-work process in sick-listed individuals due to MSD (Muskuloskeletal disorders) and WAD (Whiplash-associated disorder).

Figure 1 shows that a large proportion of individuals with MSDs returned to work between week 8 and week 26 (43% RTW). At 52 weeks, the RTW had increased to 51%, and it reached a fairly constant level of 57%. RTW for those with WAD did not increase as fast as for those with MSDs, and the RTW stabilised at a lower level. At 26 weeks, 18% had returned to work. This percentage increased to 44% two years after sick listing, and this level was maintained through the remaining follow-up period.

Table 2 describes in detail the distribution of different types of public transfer in the WAD group at six months, one year, two years, and three years of follow-up. The number of sick-listed individuals decreased from 78% at six months to 20% at two years and 8% at three years. The number of people who were sick-listed continued to decrease throughout the entire follow-up period despite the stagnation in RTW. Table 2 shows that this was due to people receiving other types of public transfer income, such as state-supplemented part-time employment (flex job)*,* disability pension, etc.

**Table 2:** **Types of transfer payment in sick-listed individuals with WAD**^**a**^

	**Week 26**	**1 year**	**2 years**	**3 years**
	**N (%)**	**N (%)**	**N (%)**	**N (%)**
Return to work	19 (18)	35 (34)	46 (44)	36 (43)
Unemployment benefit	1 (1)	3 (3)	3 (3)	0
Flex job	1 (1)	5 (5)	14 (14)	18 (22)
Vocational rehabilitation	1 (1)	4 (4)	6 (6)	2 (2)
Maternity leave	1 (1)	5 (5)	4 (4)	3 (4)
Social assistance	0	3 (3)	7 (7)	7 (8)
Disability pension	0	0	3 (3)	10 (12)
Sickness benefit	81 (78)	49 (47)	21 (20)	7 (8)
Total	104 (100)	104 (100)	104 (100)	83 (100)^b^

ªWhiplash-associated disorder.

^b^Some of the subjects entered the study too late to be followed up for the full 3 years (21 people were missing at 156 weeks).

Table 3 shows the distribution of public transfer income in the MSD group. Even though the same tendency to regroup into other types of transfer income was seen, a relatively small number of people received flex job and disability pension.

**Table 3:** **Types of transfer payments in sick-listed individuals with MSDs**^**a**^

	**Week 26**	**1 year**	**2 years**	**3 years**
	**N (%)**	**N (%)**	**N (%)**	**N (%)**
Return to work	1,369 (43)	1,638 (51)	1,807 (57)	1,400 (57)
Unemployment benefit	213 (7)	283 (9)	210 (7)	120 (5)
Flex job	14 (0)	48 (2)	217 (7)	225 (9)
Vocational rehabilitation	15 (0)	73 (2)	135 (4)	81 (3)
Maternity leave	11 (0)	28 (1)	42 (1)	27 (1)
Social assistance	48 (2)	60 (2)	124 (4)	98 (4)
Disability pension	5 (0)	20 (1)	108 (3)	135 (5)
Sickness benefit	1481 (46)	937 (29)	361 (11)	240 (10)
Other	43 (1)	96 (3)	170 (6)	145 (6)
Total	3,199 (100)^b^	3,183^c^ (100)	3,174 (100)^d^	2,470 (100)^e^

^a^Musculoskeletal disorders.

^b^5 dead or emigrated.

^c^21 dead or emigrated.

^d^30 dead or emigrated.

^e^734 dead or emigrated or censored due to late study entry.

The results of the multivariate logistic analysis are presented in Table 4. At week 26, the RTW was significantly lower in the WAD group than in the MSD group (OR for RTW0.29 ( 0.18–0.49)). The OR for RTW in sick-listed individuals with WAD increased but remained significantly lower at two years OR = 0.54 (0.36–0.81), and three years of follow-up OR = 0.53 (0.34–0.84). The results are adjusted for age, gender, and educational level.

**Table 4:** **Return to work in sick-listed individuals with WAD**^**a **^**compared to those sick-listed with MSDs**^**b**^

	**Week 26**^**c**^		**1 year**^**d**^		**2 years**^**e**^		**3 years**^**f**^	
	**OR**	**CI**	**OR**	**CI**	**OR**	**CI**	**OR**	**CI**
Model 1^g^	0.3	(0.18–0.49)	0.48	(0.32–0.72)	0.60	(0.41–0.89)	0.59	(0.38–0.91)
Model 2^h^	0.32	(0.19–0.53)	0.52	(0.34–0.78)	0.63	(0.42–0.93)	0.59	(0.38–0.93)
Model 3^i^	0.29	(0.18–0.49)	0.46	(0.30–0.71)	0.54	(0.36–0.81)	0.53	(0.34–0.84)

ªWhiplash-associated disorder.

^b^Musculoskeletal disorders.

^c^5 dead or emigrated in the MSD group.

^d^21 dead or emigrated in the MSD group.

^e^30 dead or emigrated in the MSD group.

^f^734 dead or emigrated or censored due to late study entry in the MSD group, and 21 missing due to late study entry in the WAD group.

^g^Model 1: raw data.

^h^Model 2: controlled for age and gender.

^i^Model 3: controlled for age, gender, and education.

## Discussion

In this study, individuals who were long-term sick-listed because of whiplash-associated disorder were slower and less likely to return to work than people who were sick-listed due to other types of musculoskeletal disorders. In both groups, return-to-work stabilised after two years of follow-up.

After two years 56% of WAD patients and 43% of MSD patients had not returned to work and this is a higher proportion than observed in other studies. In 2001, Kasch et al. reported that 12% of subjects had not returned to normal daily activity or had returned only to modified job functions one year after a whiplash injury [10]. One Dutch study had results in line with this finding, as 12.6% of individuals had persistent work disability after one year [11]. The low level of RTW in our cohort is primarily explained by differences in the populations. Our cohort consisted of long-term sick-listed people, while Kasch et al. included people who had been in contact with the local emergency room within two days after the trauma. Some of these people may not have been sick-listed at any time. Likewise, the Dutch study group consisted of people who had initiated compensation claim procedures and the threshold for starting such procedures is apparently low in the Netherlands [11]. The slower RTW-rate in the WAD group during the first year could be explained by the fact that the MSD group included less severe disorders such as fractures.

In a recent Danish study early classification of patients into risk strata based on biological and psychosocial functions predicted non-recovery and decreased work ability among patients exposed to whiplash [17]. Among patients in the high-risk group only 32% had returned to work after one year. In our analysis 34% had returned to work after one year, which indicates that our cohort includes individuals similar to the high-risk categories described by Kasch et al. [17]. Those still sick-listed after 8 weeks (and thereby included in our study) presumably would have had high risk scores initially. Unfortunately, we are not able to classify the cohort in the proposed risk strata to compare the results.

In Denmark, there is general access to transfer income including disability pension, sickness benefit, and unemployment benefit. A person can only receive one transfer payment at a time, and there are different time limits for most types of transfer payment. Thus, estimation of the duration of the sick leave while neglecting other types of transfer payment would underestimate the risk of an unfavourable vocational prognosis [18]. Therefore RTW was chosen as outcome, though measures of sustained RTW would have been preferred but was not possible due to the frequent switching between different types of public transfer income in Denmark.

In Figure 1 a stagnation of RTW is seen through the follow-up period and fewer individuals are sick-listed after 3 years. In Denmark, one can receive sickness benefits for one year with the possibility of extension up to two years, if there is a wait for treatment. This is one of the possible explanations for the stabilisation seen in our results after two years of sick leave. There was a tendency to regroup into other types of public transfer payments, especially flex job and disability pension in the WAD group. To be granted a disability pension in Denmark work ability has to be permanently low, and after three years, 12% in the WAD group had received disability pension, compared to 5% in the MSD group.

It has previously been reported that a large proportion of patients with WAD had been granted disability pension [9]. A study of accident victims with WAD assessed by the National Board of Industrial Injuries in Denmark found that 29% eventually received disability pension [8]. In a study among members of a Danish WAD patient society, more than 40% had been approved for disability pension [19], although the higher proportion was probably due to selection of the most disabled patients, those who would join a patient society. The evaluation of chronic low work ability is a long process, and more participants in the present study would probably have a disability pension if our cohort was followed up for more than two or three years.

The predominance of women in the WAD group was surprisingly high compared to the MSD group. In line with this, a German study found that although males were involved in a greater number of rear-impact collisions, females reported more neck distortion injuries, which indicates that females are more susceptible to whiplash injury [20]. However, on this point, the literature is inconsistent: while two systematic reviews have found evidence of an association between female gender and poor recovery after whiplash injury [21,22], one systematic review has found strong evidence that female gender is not associated with a poorer prognosis [5]. In the present study, we found no change in OR for RTW when adjusting for age and gender, but gender differences in psycho-social prognostic factors could influence the result in studies where such factors are not included in the analyses. In most studies low education is a negative prognostic factor, also in studies of WAD [22]. However the opposite effect was observed in this study, since the OR decreased after adjustment for education and we have no apparent explanation to this fact. The development of WAD and disability after whiplash is a complicated interaction between a predisposing vulnerability before the accident and multi-factorial maintaining factors after the accident [23]. In this study only the socioeconomic consequences of WAD have been studied without knowledge of vulnerability and coping factors.

The strengths of this study were the long follow-up and the possibility of following the sick-listed individuals on a weekly basis regarding RTW and other types of public transfer income through the DREAM register. However, there is a risk of assigning unemployed individuals living on their own financial resources to the group of working people. However, as discussed by Stoltenberg and Skov [12], this is probably the case for only a few people in the cohort due to the Danish welfare system.

One limitation of this study was the validity of the diagnostic label of WAD. While for some people we had self-reported diagnoses, others were diagnosed by their general practitioner and we have no further information about medical or diagnostic procedures. It was not possible to compare diagnoses given by GPs versus self-reports, and the validity of the diagnosis was not evaluated in this study.

Furthermore, additional weeks of sick leave could be due to diseases other than the first diagnosis of WAD or MSD used in this study. Thus, our results should be interpreted as being from a study of the RTW process in people with WAD as the initial diagnosis in a follow-up study of attachment to the labour market. Since the results show stabilisation in RTW after two years, the lack of three years of follow-up in the entire cohort appears to have had minor consequences for the overall result of this study. It would have been interesting to include some of the prognostic factors for a chronic course of WAD in the multivariate analyses, but, unfortunately we did not have that kind of information in the cohort.

## Conclusions

This study showed that work disability is a common problem among individuals who are registered as having whiplash associated disorder as a cause of their long-term sick leave. The RTW process is slower and the RTW rate is lower among individuals sick-listed with WAD compared to individuals sick-listed with other MSDs. These findings suggest that an active rehabilitation is important to sick-listed individuals with WAD at an early stage of the process, and there is a need for future intervention studies with early onset of treatment for people with WAD to prevent chronic working disability.

## Competing interests

The authors declare that they have no competing interests.

## Authors’ contributions

PS and AM initiated this study and CS was responsible for all analyses of data. SB made the first draft of the manuscript and JWH, PS, AM and CS contributed to the interpretation of data and all authors were involved in the revision of the manuscript. All authors read and approved the revision and the final manuscript.

## Pre-publication history

The pre-publication history for this paper can be accessed here:

http://www.biomedcentral.com/1471-2458/14/113/prepub
